# Direct Ink Writing and Photocrosslinking of Hydroxypropyl Cellulose into Stable 3D Parts Using Methacrylation and Blending

**DOI:** 10.3390/polym17030278

**Published:** 2025-01-22

**Authors:** Mehmet-Talha Yapa, Gopakumar Sivasankarapillai, Jacques Lalevée, Marie-Pierre Laborie

**Affiliations:** 1 Chair of Forest Biomaterials, Institute of Earth and Environmental Sciences, Faculty of Environment and Natural Resources, University of Freiburg, Werthmanstr. 6, 79085 Freiburg im Breisgau, Germany; mehmet.talha.yapa@biomat.uni-freiburg.de (M.-T.Y.); gopakumar.sivasankarapillai@fmf.uni-freiburg.de (G.S.); 2 Freiburg Materials Research Center, Stefan-Meier-Straße 21, 79104 Freiburg im Breisgau, Germany; 3 Université de Haute Alsace, CNRS, IS2M UMR 7361, 68100 Mulhouse, France; jacques.lalevee@uha.fr; 4 Strasbourg University, 4 Rue Blaise Pascal, 67000 Strasbourg, France; 5 Strasbourg University, CNRS, Institut Charles Sadron UPR22, 23 rue du Loess, 67000 Strasbourg, France

**Keywords:** hydroxypropyl cellulose, methacrylate grafting, photocrosslinking kinetics, rheology, 3D printing, direct ink writing, polymer blends

## Abstract

Two 50% solid content solutions of methacrylated hydroxypropyl cellulose (MAHPC) with respective substitution degrees of 1.85 ± 0.04 (L_MAHPC) and 2.64 ± 0.04 (H_MAHPC) were screened for rheological properties, photocrosslinking kinetics and printability in relevance to direct ink writing (DIW). Photo-rheological and printability studies reveal that the rheological properties of both MAHPC inks are better suited for DIW than those of hydroxypropyl cellulose (HPC) inks. Namely, methacrylate grafting improves shear dynamic moduli at low strain but also shear thinning and shear recovery. Both inks completely cure within 30 s upon shining UV light. Photocrosslinking is found to follow the phenomenological autocatalytic Sestak–Berggren kinetic model. However, prolonged exposure to UV light past full cure upon DIW leads to part fracture. The narrow UV-cure time window consequently precludes the production of multilayer parts using UV-assisted DIW for these neat MAHPC inks. In contrast, when blending MAHPC with HPC, an optimal balance between curing kinetics and DIW conditions is achieved, and stable, high-fidelity 150-layered parts are produced. Altogether this research highlights the need to design the content of photocrosslinkable moieties of cellulose derivatives to photoprint high fidelity and stable 3D parts from HPC inks.

## 1. Introduction

Within the pursuit of sustainability, 3D printing of wood-based polymers is attracting increasing attention due to the renewable and CO_2_-neutral nature of wood polymers and the material efficiency of additive manufacturing [1,2,3,4,5,6]. Natural polysaccharides, their derivatives and blends thereof have all been implemented in some type of additive manufacturing technology, delivering 100% bio-based parts in some cases [7,8,9,10,11,12,13,14,15]. For direct ink writing, an extrusion-based process occurring at room temperature, feedstock choices have naturally converged onto cellulose derivatives and nanocellulose due to their solubility and/or propensity to form colloidal suspensions in solvents [1,12,16,17,18,19,20,21].

Along this direction of 3D printing with wood-based materials, our research group has recently embarked on the goal of developing “lignin-inks” that are fully based on wood–polymers and have high lignin content (at least 50%) for direct ink writing [22]. Inspired by the plant secondary cell wall morphogenesis, we combine a chiral liquid crystalline cellulosic derivative, such as a cellulose ether or ester with organosolv lignin. In this approach, the lyotropic cellulose derivative serves as a shear-thinning agent during extrusion and as a structuring agent for the 3D part. On the other hand, lignin consolidates the assembly under quiescent conditions on the printing platform thereby imparting integrity and shape fidelity to the 3D part [22]. While this concept has been demonstrated with multiple cellulose derivatives [22,23,24,25] and with cellulose nanocrystals [26] the consolidation effect of lignin appears to be mainly physical and is insufficient to warrant long-term stability to the 3D parts. To enhance part integrity and stability, further efforts have, therefore, focused on implementing additional chemical crosslinking on “lignin inks” via condensation chemistry [23] or photochemistry [27]. The simple addition of photocrosslinkers to “lignin inks” has been shown to induce substantial photocrosslinking [27] but it is still insufficient to warrant long-term stability for centimeter-large 3D parts. Grafting photocrosslinkable moieties on the components of “lignin inks” might, therefore, help enhance reactivity to light and the long-term stability of centimeter-large 3D parts.

The literature abounds in successes in imparting photocrosslinkability on both cellulosics and lignin using chemical grafting of photocrosslinkable moieties [28]. In particular, the rapid photocure of acrylic-based chemicals [29] has been exploited for UV curing of cellulosic polymers [30,31]. It has been successfully applied to cellulose nanocrystals [32], cellulose nanofibers [33], carboxymethyl cellulose [34,35,36], cellulose diacetates [37] and Hydroxypropyl cellulose (HPC) [38,39,40,41,42,43,44,45,46]. For example, Rothmanner et al. [37] developed photocurable resists from methacrylated cellulose diacetate, enabling the fabrication of cellulosic architectures with resolutions below 1 µm and feature sizes under 500 nm with two-photon direct laser writing. Soulard and Jean also acrylated cellulose nanocrystals to prepare inks for stereolithography [32]. Walters et al. [38] explored a blend of methacrylated hydroxypropyl cellulose (MAHPC) and cellulose nanocrystals to develop a film with tunable optical and mechanical properties. Other studies have applied UV-curable MAHPC for various biotechnological applications, including cell-release [40], tissue engineering [40], multilayer cell culture systems [44,45] and articular cartilage repair [45]. Marsano et al., meanwhile, focused on the phase [47] and gel behavior [46] of MAHPC. Qi et al. [42] showcased its application in photolithography. MAHPC has also proven useful for photocrosslinking and DIW blends of HPC and chitosan [39]. Vignolini’s group demonstrated that a low extent of methacrylation on low molecular weight HPC enabled the DIW and photocuring of small photonic objects [43].

With the long-term objective of developing in situ photocrosslinkable lignin/HPC inks for DIW, this study investigates the impact of grafting methacrylic moieties onto HPC with respect to rheology, photocrosslinking kinetics and DIW printability. This study further aims to formulate suitable HPC-based inks to manufacture centimeter-large 3D parts with good shape fidelity and stability.

## 2. Materials and Methods

### 2.1. Raw Materials

Hydroxypropyl cellulose with a hydroxypropyl content ranging from 53.4% to 80.5% and a molecular weight of 100,000 g/mol as per supplier technical datasheet was purchased from Thermo Fisher GmbH (Kandel, Germany) [48]. We determined a degree of substitution (DS) of 2.4 and a molar substitution (MS) of 4.76 for this HPC (Figure S2). HPC was oven-dried at 100 °C for 3 h and stored in a desiccator with Silica gel orange (particle size: 2–5 mm) and an indicator from Carl Roth GmbH, (Karlsruhe, Germany) for an additional 24 h to ensure thorough drying. Moisture content was not specifically determined. Methacrylic anhydride (MA), with a purity of ≥94% and containing 2000 ppm of topanol A inhibitor, the catalyst 4-(Dimethylamino)-pyridine (DMAP) with a purity of ≥99.0% for the grafting reaction were obtained from Sigma-Aldrich (Taufkirchen, Germany). For the UV-initiated photocrosslinking, the radical initiator, phenyl bis(2,4,6-trimethylbenzoyl)-phosphine oxide with a purity of ≥96.0%, was procured from TCI Deutschland GmbH in Eschborn, Germany. p-Benzoquinone was selected as an inhibitor due to its oxygen-insensibility and purchased from Sigma-Aldrich (Taufkirchen, Germany). Acetone (≥99.5%), ethanol (≥99.8%), and glacial acetic acid (≥99.7%) were acquired from VWR International GmbH (Darmstadt, Germany). Sodium hydrogen Ecarbonate (NaHCO_3_, ≥98.5%) from Thermo Fisher GmbH (Kandel, Germany) was utilized for the neutralization after grafting. Methacrylic acid (MAA) was purchased from Thermo Scientific Chemicals (Geel, Belgium) and used as a reference for Fourier Transform Infrared analysis. Sodium Methacrylate was acquired from TCI Deutschland GmbH in Eschborn, Germany for FTIR analysis. For NMR analyses, Chloroform-d1 and methanol-d4 were obtained from Euriso-Top (Saint-Aubin, France) and Chromium(III) acetylacetonate (Cr(acac)_3_) was purchased from Sigma-Aldrich, Taufkirchen, Germany.

### 2.2. Methods

#### 2.2.1. Synthesis and Characterization of Methacrylated HPC

##### Synthesis of Methacrylated HPC

**Scheme 1:**
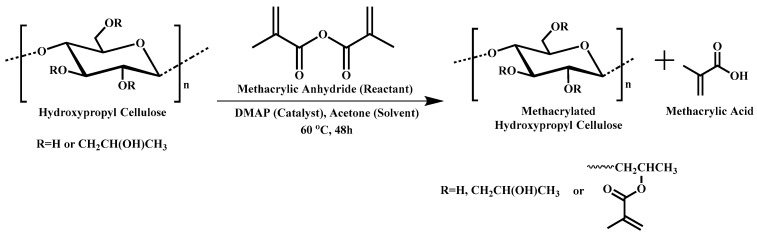
Reaction scheme for methacrylic grafting onto HPC.

HPC was functionalized with MA (Scheme 1) following the reported protocols with minor modifications [41,42]. The MA:AGU molar ratio was selected as 1.43:1 to produce a methacrylated hydroxypropyl cellulose with a low degree of methacrylation (L_MAHPC) and as 4.29:1 to obtain a high degree of methacrylation in the HPC (H_MAHPC). For both reactions, the MA:DMAP molar ratio was fixed at 15.85:1.

In a typical grafting reaction, 5 g of HPC and the required amount for DMAP catalysts were homogeneously dissolved in 75 mL of acetone with magnetic stirring in a 100 mL round-bottom flask equipped with a condenser. The flask was placed in a silicone oil bath maintained at 60 °C, and MA was introduced dropwise in the required amount. The total reaction time was 48 h. Upon completion, the reactor was allowed to cool to room temperature in air. The reaction products were immediately neutralized with NaHCO_3_ using a Sartorius PB-11 pH Meter (Göttingen, Germany) equipped with an electrode from Xylem SI Analytics BlueLine 15 pH NTC10 (Weilheim, Germany). It was then soaked for three hours under magnetic stirring in ca. 500 mL of distilled water. Further washing of the neutralized product occurred multiple times in a vacuum filter with excess water. The neutralization step with NaHCO_3_ was repeated for a duration of 24 h. The product was finally filtered and washed with excess water to purify MAHPC species and freeze-dried for storage. Storage occurred in the dark at 6–8 °C. The products were analyzed within one week of synthesis.

##### Determination of Molecular Structure of MAHPC

The molecular structure of the HPC and MAHPC was characterized using FTIR and NMR spectroscopies. FTIR spectra were acquired on triplicate specimens on an FTIR spectrometer 65 (PerkinElmer, Shelton, CT, USA) operating in attenuated total reflection (ATR) mode with a ZnSe crystal. In total, 32 scans were acquired from 650 to 4000 cm^−1^ at a resolution of 4 cm^−1^. For semi-quantitative analysis, spectral intensities were normalized to the reference stretching vibration of the β-D-glucopyranose ring C-O-C bond at 1050 cm^−1^ [49,50]. FTIR spectra of MA, MAA, sodium methacrylate, and DMAP were also acquired as controls.

^1^H-NMR spectra were acquired on polymer solutions, 10 mg/mL in deuterated chloroform on a Bruker Avance III HD 400 MHz spectrometer equipped with a BBFO probe at 298 K. A relaxation delay of 3 s was selected and 64 scans acquired [51].

For ^13^C-NMR analysis, 30 mg of polymer was dissolved in 0.6 mL of deuterated methanol to which Cr(acac)_3_ (2.5 mg) was added as a relaxation agent and placed in a 5 mm OD NMR tube for measurement. ^13^C spectra were acquired on a Bruker Avance IV Neo 400-MHz spectrometer equipped with a BBFO probe at 298 K. The 90° pulse for ^13^C-NMR was set at 8.0 µs, the relaxation delay at 2 s, and data were acquired over 3000 scans [52].

#### 2.2.2. Formulation and Characterization of HPC and Photocrosslinkable MAHPC Inks

##### Formulation of HPC and Photocrosslinkable MAHPC Inks

To determine the most potent solvent for the ink formulations, HPC, MAHPC, SpeedCure BPO, and p-Benzoquinone each underwent solubility testing in acetone, ethanol, acetic acid according to published protocols [53]. Ethanol appeared as a universal solvent for all components and was used to prepare the polymeric inks. Based on prior work [23,25,27,54] the polymeric inks were prepared to reach a solid content of 50% and comprised 1 wt% SpeedCure BPO and 0.01% p-Benzoquinone, with respect to polymer mass. The inks were stored in the dark at room temperature for three days to enhance miscibility prior to use.

##### Monitoring the Crosslinking Kinetics of HPC and Photocrosslinkable MAHPC Inks

The impact of shining UV light on the rheological properties of the inks was tested on an MCR 301 rheometer operating in parallel plate geometry at a strain of 0.01% and a frequency of 10 rad/s. To enable UV light irradiation, the fixture consisted of standard steel top plate, 25 mm diameter on the top, and of a glass plate (PTD120/UV, 65 mm diameter, Anton Paar, Graz, Germany) on the bottom onto which 100% UV light was uniformly shun from a 30 cm distance starting at t = 100 s. After placing the sample in the parallel plate fixture, sealing was carried out with Krytox GPL 105 mineral oil (Chemours, Wilmington, DE, USA) and another layer of Vaseline. The UV light (39,000 mW/cm²) was generated from a UV-LED Spot P light source (Opsytec Dr. Gröbel GmbH, Ettlingen, Germany) operating at a wavelength of 395 nm. The shear storage modulus, G’(t) was monitored from the onset of light exposure considered as t = 0 and used to follow the degree of mechanical cure (β) [55,56]:

(1) $$\mathrm{mechanical}\;\mathrm{degree}\left(ß(t)\right)=\frac{G’\left(t\right)-G’(0)}{G’\left(\infty \right)-G’(0)}$$

where G′ (0) denotes the storage modulus at t = 0, G′(t) the storage modulus at a time t past onset of UV light shining and, G’(∞) the storage modulus at its maximum, asymptotic value. 

#### 2.2.3. DIW of HPC-Based Inks

##### Screening the Printability of HPC and MAHPC Inks for DIW

Screening of printability for HPC and MAHPC inks followed the Paxton protocol [57]. The inks were first centrifuged at 4500 rpm for 30 min at 25 °C in 30 cc plastic cartridges (Nordson EFD, Feldkirchen, Germany) and then manually extruded through 0.41 mm diameter plastic conical needle tips (Nordson EFD, Feldkirchen, Germany). Fiber formation and layer stacking upon manual extrusion were visually assessed.

Shear thinning and modulus recovery behaviors were also examined on an MCR 301 rheometer (Anton Paar, Graz, Austria) operating in the dark with the same parallel plate geometry and sample preparation as previously described. Shear thinning was monitored by measuring rotational shear viscosity from 0.1 to 100 s shear rates. Storage modulus recovery was assessed by comparing the G’ values at 0.01% strain before and after a 200 sec period of straining at 500% [58,59]. It was ensured through amplitude sweep tests that the selected strain of 0.01% was below the linear viscoelasticity limit.

(2) $$\mathrm{Recovery}\left(\%\right)=\frac{G’\;\mathrm{at}\;600\;s}{G’\;\mathrm{at}\;200\;s}\times 100$$

##### Direct Ink Writing and Properties of 3D Parts

The neat HPC and MAHPC inks were transferred to 30 cc plastic cartridges (Nordson EFD, Feldkirchen, Germany) and centrifuged at 4500 rpm for 30 min at 25 °C. A cylinder, 2 cm in diameter, was printed from 0.41 mm tapered plastic needles (Nordson EFD, Feldkirchen, Germany) using a fourth-generation Envisiontec 3D-Bioplotter Developer Series (Gladbeck, Germany) operating at 5 mm/s with a printing pressure ranging from 2.5 to 3 bar. The printing platform was uniformly illuminated with 100% light intensity (39,000 mW/cm²) under UV light generated with a UV-LED Spot P (Opsytec Dr. Gröbel GmbH, Ettlingen, Germany) (Figure S3).

To assess the gel content achieved during the UV-assisted DIW, single-layer rectangular specimens (30 × 10 × 3.2 mm^3^) were also printed. For a monolayer the exposition to UV light lasted one minute. After thorough drying for 3 days in the open air and another 24 h in a desiccator containing silica gel, the specimen’s gel content was determined in triplicates. The dry specimens were first weighted on an ABT 100-5M analytical balance (KERN & SOHN GmbH, Balingen, Germany) and then immersed for 24 h in ethanol. Their residual mass was measured again after thorough washing and drying for 24 h at 100 °C. Gel content was then computed as:

% crosslinked mass = 100 × (residual dry mass of photocured samples after solvent extraction/initial dry mass of photocured samples) (3)

The photocrosslinkable ink formulation was further optimized by printing 50/50 and 25/75 %*w/w* blends of HPC and MAHPC in the tallest possible cylinders and examining part quality and shape fidelity as:

(4) $$∆X\%=\frac{\left|X_{\mathrm{printed}}-X_{\mathrm{theorical}}\right|}{X_{\mathrm{printed}}}\times 100$$

where X_theoretical_ refers to all dimensions (x,y,z) as designed in the Perfactory software (Version 3.2.3594.1909), while X_printed_ denotes all dimensions measured at five different points on the freshly printed part. Additionally, shape stability was visually monitored after one week of storage under ambient conditions.

Dried HPC, MAHPC, and their photocrosslinked variants were analyzed using Thermogravimetric Analysis (TGA) on a Pyris 1 TGA (PerkinElmer, Shelton, CT, USA) operating at 10 °C per minute from 25 °C to 900 °C under nitrogen. Each sample weighed approximately 5 mg, and the analysis was performed three times for each sample.

## 3. Results and Discussion

### 3.1. HPC Methacrylation Can Be Tuned from the Conditions of the Grafting Reaction

**Figure 1:**
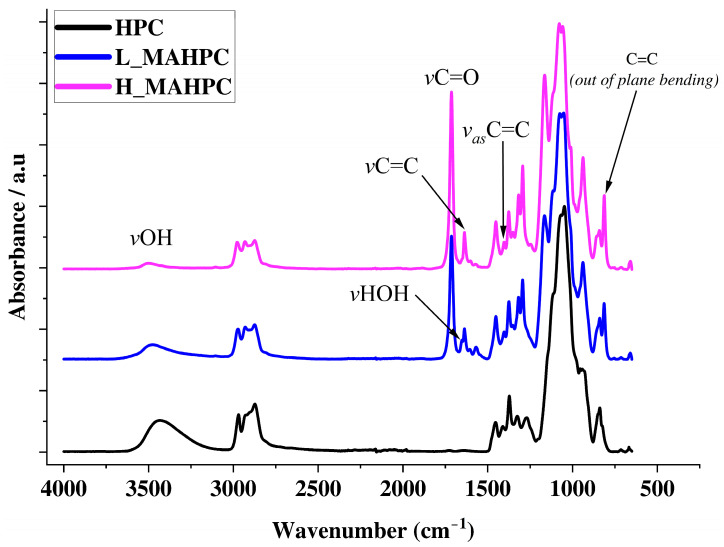
FTIR spectra of HPC and methacrylated HPCs.

The FTIR spectra of HPC and MAHPCs reveal a significant decrease in the hydroxyl (OH) stretching vibration at 3433 ± 2 cm^−1^ upon methacrylation and a shift to higher wavelengths at 3478 ± 4 cm^−1^ for L_MAHPC and at 3506 ± 5 cm^−1^. for H_MAHPC (Figure 1). Expectedly, a carbonyl (C=O) stretch appears at approximately 1720 cm^−1^, along with CH₂=C vibrations at 1630–1640 cm^−1^ (stretching), ~1400 cm^−1^ (scissoring), and ~810 cm^−1^ (out-of-plane bending) [60]. A minor shoulder around 1650 cm^−1^ likely indicates the HOH-bending vibration of water. Importantly, these signals do not arise from residual MA, MAA, or sodium methacrylate (Figure S1), but rather reflect successful methacrylation. Each of these vibrational intensities increases in proportion to the molar ratio of MA, suggesting that FTIR can distinguish MAHPC of distinct degrees of methacrylation.

**Figure 2:**
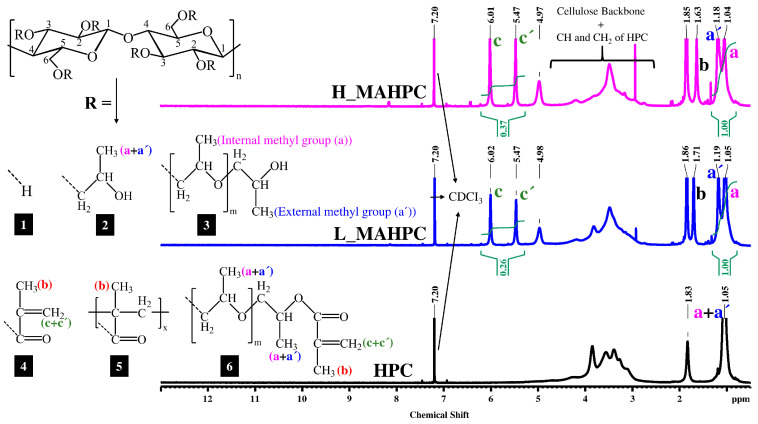
^1^H-NMR spectra of HPC and methacrylated HPCs.

Upon methacrylation of HPC, several substitution patterns of the HPC hydroxyl groups can theoretically be envisioned, including a hydrogen (1), a single hydroxypropyl unit, (2) multiple hydroxypropyl units (3) a methacryloyl unit (4) multiple methacryloyl units (5), and a methacrylated hydroxypropyl unit (6) (Figure 2). ^1^H-NMR enables a deeper understanding of substitution patterns of MA on HPC. It also enables quantitative determination of the MAHPC methacroyl content (Figure 2).

The HPC ^1^H NMR spectrum exhibits the typical resonances reported in the literature: the methyl hydrogen (CH₂CH(OH)C**H_3_**) is expectedly detected at 1.05 ppm (a+a’) [39,61,62,63,64] while cellulosic backbone protons appear in the 2.5 to 5 ppm range together with some hydroxypropyl protons [61,62]. An unexpected chemical shift at 1.8 ppm in HPC and in its methacrylated derivatives has no precedent in the HPC literature but is seen in modified derivatives [42]. It may reflect a methine resonance [65] or a shifted OH signal.

Upon methacrylation alkene protons from the (C**H_2_=**C(CH_3_)CO) expectedly appear at 6.0 and 5.5 ppm (c) [39,41,42,45,64]. A sharp chemical shift also appears at 1.7–1.6 ppm (b). It likely stems from the methyl group nearest to the methacryloyl unit (CH_2_=C(C**H_3_**)CO) [39,41,42,45,64]. A chemical shift at 5.0 ppm corresponding to the methine proton of the hydroxypropyl unit attached to a methacrylate group confirms preferential grafting onto the hydroxypropyl moiety of HPC. New resonances of weak intensity also appear around 1.5 ppm, possibly reflecting some minor degree of MA polymerization. The absence of a (CH_2_=C(CH3)COO**H**) proton at 12 ppm indicates that MAA has been fully removed [66], so that the observed spectral changes can only be ascribed to methacrylate grafting onto HPC. The strong resonance for the methacryloyl proton at 5.3–6.1 ppm can be used to determine the degree of methacrylation (Figure 2). The degree of methacrylation was found to increase from 1.85 ± 0.04 for L_MAHPC to 2.64 ± 0.04 for H_MAHPC (Figure S2) Also note that the broad resonance for the methyl proton of neat HPC at 1.1 ppm has evolved into two distinct resonances for internal and external methyl groups, allowing to confirm the DS of the HPC itself (Figure S2). Finally, the sharper signals of the cellulose backbone upon MA grafting are reminiscent of those in hydrolyzed cellulose derivatives [67], which could indicate some degree of chain cleavage upon grafting MA onto HPC.

### 3.2. Photocrosslinking Kinetics of MAHPC Formulations Doped with Initiators

**Figure 3:**
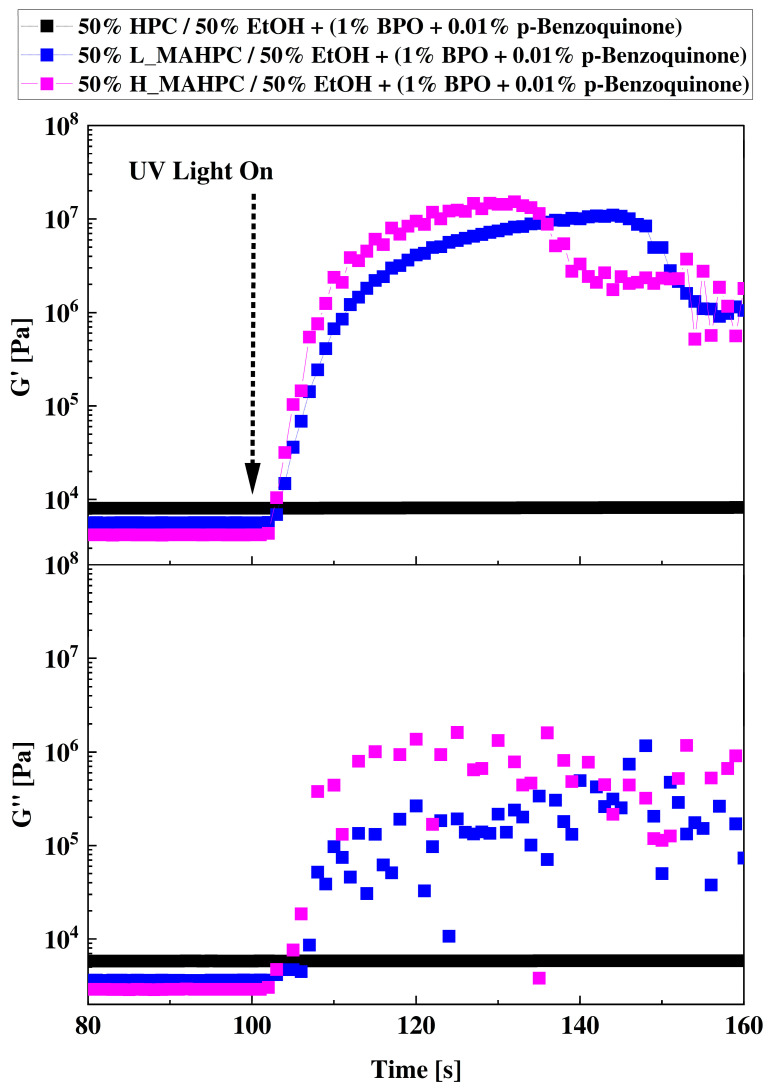
Evolution of dynamic shear moduli of HPC and MAHPC inks upon exposure to UV light in a small oscillatory rheometer.

The kinetics of photocrosslinking was monitored with photo-rheology. Prior to crosslinking the MAHPC inks exhibit the solid-like behavior of a gel with G′ > G″. Within 30 s of UV irradiation, G′ and G″ rise significantly, over 3 decades in magnitude, revealing effective photocrosslinking (Figure 3). This behavior contrasts with the light insensitivity of the HPC ink. Rapid crosslinking in MAHPC is consistent with prior work on MAHPC [43]. Following G’ rise to a plateau, a small and gradual decrease in G’ is observed. Visual inspection of the sample also reveals macroscopic fracture (Figure S4). Too long of an irradiation time past full cure likely leads to sample degradation.

**Figure 4:**
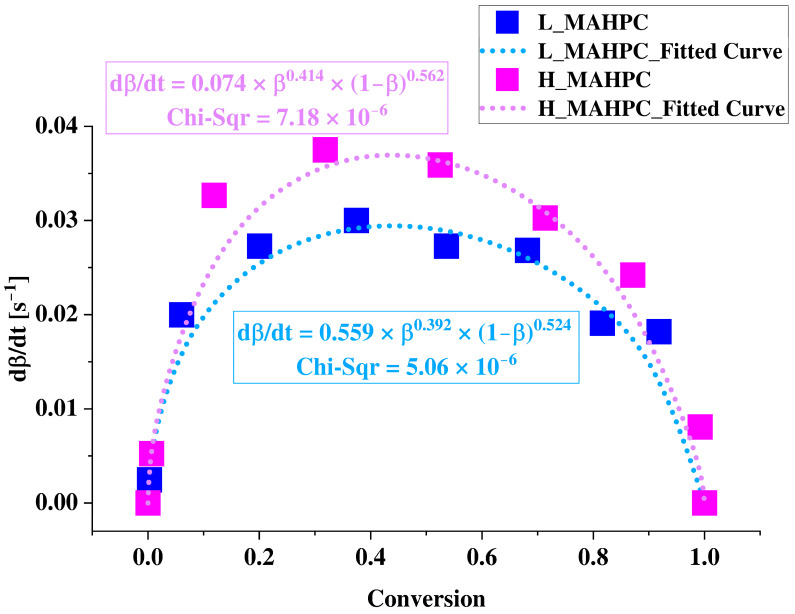
Rate of mechanical cure as a function of mechanical conversion during photocrosslinking of MAHPC inks.

Assuming 100% conversion at the shear modulus plateau, the degree and rate of mechanical conversion (ß (t)) were computed as a function of time, and the cure kinetics was modeled. Autocatalytic models have been reported to phenomenologically describe photopolymerization kinetics including photo crosslinking in acrylate systems [68,69]. Among the autocatalytic models, the Sestak–Berggren has been repeatedly used for solid-state reactions [70,71,72] and photoreactions [73,74,75,76]. Consequently, the Sestak–Berggren empirical model was selected to model the degree of mechanical conversion (ß(t)) [56] as follows:

(5) $$\frac{dß}{dt}=k\left(T\right)\times f\left(ß\right)=k\left(T\right)\times {\left.{ß}^{m}\times (1-ß\right)}^{n}$$

where n is the reaction order, m is the autocatalytic exponent, k is the reaction rate constant and dß/dt is the reaction rate (s^−1^). The reaction rates of both MAHPCs were indeed well described by the Sestak–Berggren empirical model (Figure 4). Also, note that the m and n values are <1 as recommended for autocatalytic models in the ASTM E2070-13 [77] and sum up to a total reaction order approaching 1. With the maximum reaction rate occurring at 30–35% conversion for both MAHPCs, autocatalytic kinetics are confirmed. Expectedly, the H_MAHPC exhibits slightly greater reactivity and faster reaction rates than the L_MAHPC (Figure 4).

### 3.3. Screening of HPC and MAHPC Photocrosslinkable Formulations for 3D Printing

**Figure 5:**
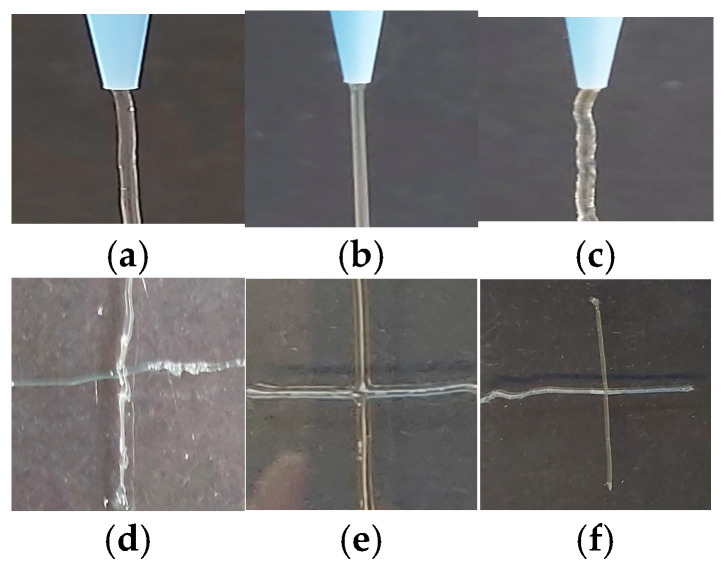
Initial 3D-printability assessment of HPC and MAHPC inks a (**a**,**d**): HPC, (**b**,**e**): L_MAHPC, (**c**,**f**): H_MAHPC; Top: fiber/droplet formation essay, Bottom: layer stacking/merging essay.

HPC and MAHPC inks all exhibit evident fiber formation and layer stacking, suggesting their potential printability in DIW (Figure 5). However, a close visual examination suggests that fiber formation and layer stacking are optimum with the L_MAHPC ink.

**Figure 6:**
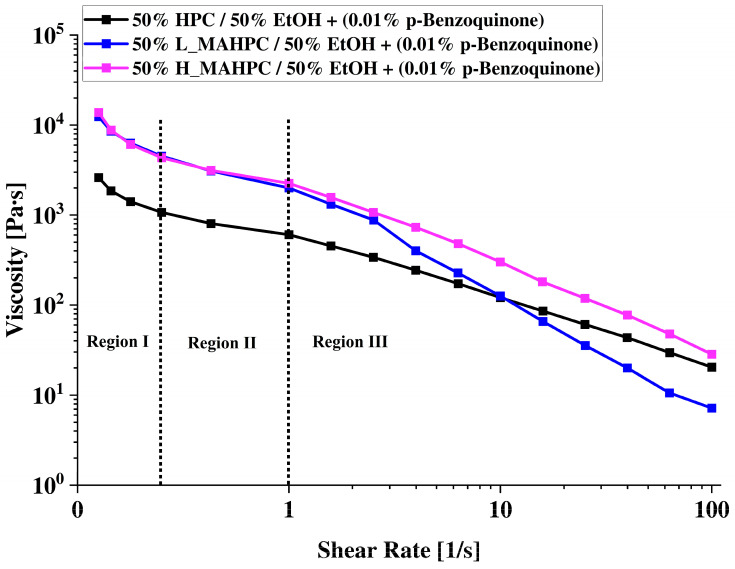
Shear-rate dependency of shear viscosity of HPC and MAHPC inks.

Rotational rheology provides a deeper insight into the impact of MA grafting on the inks’ rheological properties and printability, despite sample slippage from the fixtures at the highest shear rates [78,79] (Figure 6). First, the zero-shear rate viscosity increases significantly (4-fold) with MA grafting, as previously observed [43] (Figure 6). This might partly stem from the potential of methacrylate groups to promote stronger H-bonds through its ketone oxygen, generating a stronger, more structured gel. Also note the shear shinning of all three inks, displaying three domains of shear rate dependency for viscosity with (i) an initial strong shear thinning behavior (region I), followed by (ii) a near Newtonian behavior (region II) and finally (iii) another strong shear thinning behavior (region III). This behavior is consistent with the observations of Chan et al., 2022 [43], who proposed a distortion-induced alignment of MAHPC upon increasing shear rate. Note, however, that the zero shear rate viscosity is one to two orders of magnitude higher than that reported in Chan`s study [43]. The HPC used in Chan’s study had a significantly lower molecular weight and its MA content was also lower (DS of 0.13). In region III (Figure 6), shear thinning is significantly more marked after MA grafting. Surprisingly, however, L_MAHPC has the strongest shear thinning of all inks. Its gel structure at rest, therefore, appears to be most easily disrupted upon shearing. We will return to this observation in the next paragraph. In DIW inks, high zero-shear rate viscosity and high network strength are conducive to higher mechanical properties and higher resolution in the final 3D part [80]. At the same time, high shear thinning is conducive to flow during extrusion-based processing [80]. The optimum rheological properties are, therefore, observed for L_MAHPC, suggesting that L_MAHPC inks might be best suited for DIW, followed by H_MAHPC inks. This is consistent with the visual observations from the printability screening that L_MAHPC inks form the most uniform fibers and flawless layer stacking.

**Figure 7:**
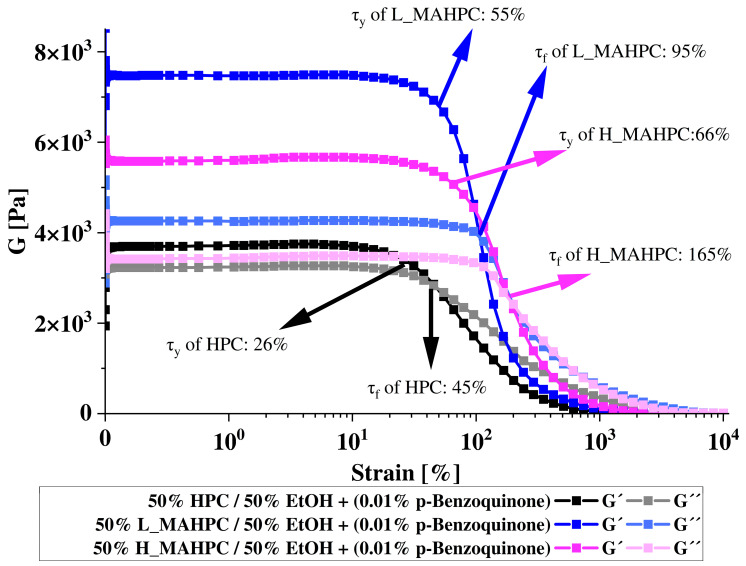
Shear storage and loss moduli of HPC and MAHPC inks from amplitude sweeps conducted at 10 rad/s (τ_y_, yield point; τ_f_, flow point).

Further strain sweeps confirmed a behavior of Bingham fluids (G′ > G″) within the linear viscoelastic range for all inks with both G′ and G″ increasing with MA grafting (Figure 7). The viscoelastic solid behavior of the inks remains up to large strains or yield points (τ_y_) (HPC: 26 ± 5% < L_MAHPC: 55 ± 7% for < H_MAHPC: 66 ± 4%) revealing remarkable stability of the structured fluid, especially after MA grafting. This contrasts with Chan’s results [43], who reported a liquid-like behavior (G′ < G″). The significantly lower molecular weight HPC (40,000 g/mol) used in Chan’s study might limit entanglement and explain the absence of gel formation [43].

MA grafting clearly accentuates the solid-like behavior, exhibiting an even larger difference in G′ and G″ and a 2-to-3-fold increases in yield point depending on MA grafting. Although both MAHPC expectedly have higher G′ and G″ at low strain than neat HPC, we note again that L_MAHPC has higher G′ and G″ than H_MAHPC. Similarly to the trend observed for shear thinning, gel moduli are not directly proportional to MA content; rather the mildly methacrylated MAHPC delivers inks of higher shear thinning and higher dynamic shear moduli than the highly methacrylated MAHPC. Considering the methacrylic substitution degree and the magnitude and strength of H-bonding in each ink might partly explain this trend: in HPC, three OH per AGU engage in intra- and inter-molecular H-bonds. In L_MAHPC (DS = 1.85), 62% of the OH groups are replaced with the methacryloyl group; there are thus about as many H-bonds as in HPC but these are stronger due to the higher electronegativity of the carbonyl oxygen of the methacryloyl moiety [81]. Intra- and inter-molecular energy might thus be higher in L_MAHPC than in HPC giving rise to higher moduli than for HPC. In H_MAHPC (DS = 2.64), 88% of OH groups are replaced with MA containing C=O groups, leaving only 12% of residual OH. In this case, the depletion of OH groups largely reduces the possible number of H-bonds.

Past the yield point, G′ and G″ are highly strain-dependent and the fluid eventually reaches a flow point (G′ = G″), after which it behaves as a viscoelastic fluid. In agreement with the trend previously observed in shear thinning, the strain dependency of dynamic moduli is more marked upon MA grafting, with the L_MAHPC being most significantly strain-dependent. However, MA-grafting increases the flow points (τ_f_) of HPC in proportion to the MA content. This confirms that MA grafting significantly contributes to the structure of the ink, delaying yield and flow point. Above the flow point, the viscoelastic properties of all three fluids appear to merge. Altogether, these rheological properties suggest that grafting MA on HPC affords more favorable attributes for DIW and part stability upon printing than HPC by enhancing moduli at low strains while still affording flow at high strains.

**Figure 8:**
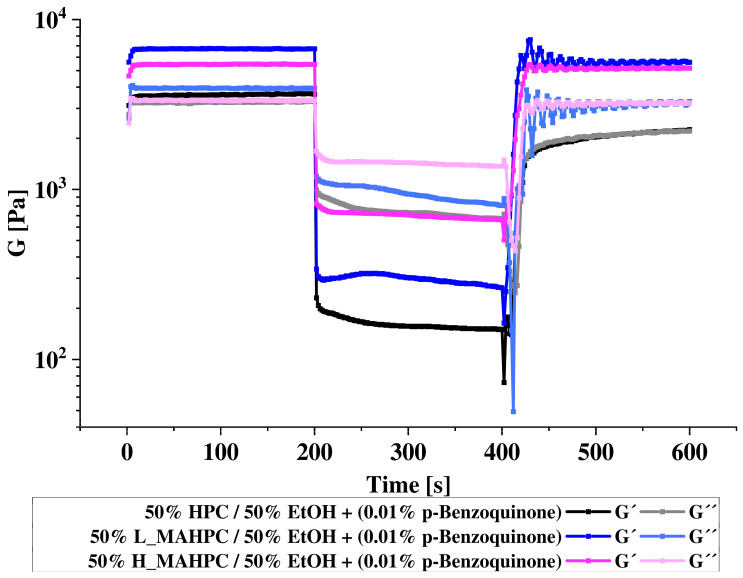
Shear storage and loss moduli of HPC and MAHPC inks during a 3-segments recovery test where segments 1 and 3 operate within the LVE at 0.01% and segment 2 operates within the flow strain region at 500%.

We then studied the recovery of viscoelastic properties when successively measured at a low strain within the linear viscoelastic range (LVE), then at a high strain within the flow region, and finally at the initial low LVE strain again. In agreement with the strain sweep results, the L_MAHPC outperforms the H_MAHPC in both shear moduli (Figure 8). MA grafting also largely enhances the recovery of G’ after high shearing, which increases from 61 ± 2% for HPC to 83 ± 1% for L_MAHPC and even 95 ± 2%. for H_MAHPC (Figure 8). The significant improvement in shear recovery reveals a stronger capacity for restructuring upon return to quiescent conditions with increasing MA content. It is especially remarkable with H_MAHPC. The flow-induced loss of structure is, therefore, particularly reversible when HPC is grafted with MA, suggesting the ability to rapidly reestablish the intermolecular interactions responsible for cohesion and solid-like behavior in the grafted HPC. While recovery portrays the reversibility of interactions, moduli portray the overall strength of these interactions. In prior work with lower MA grafting (DS of 0.13), the viscosity was found to be even higher after fast shearing, presumably due to higher ordering upon recovery from the high shear [43].

In summary, HPC and MAHPC samples all appear well amenable to DIW from the rheological standpoint and MA grafting clearly improves the ink rheological properties for DIW.

#### DIW of HPC and Photocrosslinkable MAHPC Inks

HPC inks with and without initiators are easily printable but undergo limited photocrosslinking in the absence of photocrosslinkable functional groups [27]. Therefore, only small and soft structures can be printed. In prior work, the addition of lignin has been found pivotal in limiting HPC compliance and consolidating the printed part [54]. In the absence of a consolidating polymer such as lignin or crosslinking, the neat HPC retains a high compliance and propensity to deform. As reported by Chan et al. [43], the grafting of MA onto HPC should afford rapid photocrosslinkability and thus limit compliance and propensity to deform, while maintaining the printability [43]. Upon attempting to DIW and photocrossling MAHPC formulations on the printing platform we observed that photocrosslinking was almost instantaneous and resulted in fracture of the 3D part (Figure S4). Fracture likely stems from cure stresses generated during the volumetric contraction induced by the rapid crosslinking. Fracture might also arise from polymer degradation upon prolonged exposure to UV past the full cure point as revealed from the photo-rheology results. Thus, while the neat HPC lacks photocrosslinkability, the MAHPC with its relatively high DS in comparison to prior work [43] photocrosslinks at too fast of a rate to enable prototyping 3D parts. In Chan’s work [43], the aqueous MAHPC ink exhibited significantly lower methacrylation DS (by one order of magnitude) and higher solid content. Additionally, crosslinking was temporally staged on the platform so as to enable relaxation of LC structuration within the moist and soft printed part prior to full cure. As our work aims at developing formulations for prototyping with an immediate cure of the part upon printing, a staged cure cannot be envisioned. We therefore did not attempt to further print and photocrosslink multilayer parts from pure MAHPC. Rather we explored MAHPC/HPC blends as a means to tune crosslinking kinetics to the DIW processing conditions. Solution polymer blends were, therefore, formulated as inks with 50% solid content and H_MAHPC/HPC *w/w* ratios of 25/75 and 50/50.

Blending HPC with H_MAHPC inks indeed provides an avenue to print intact structures with as many as 150 layers (Table 1). This is the tallest structure that could be printed with HPC based blends so far [27]. The blend inks are, therefore, well amenable to produce multilayer objects of a few centimeters in height. Note, however, cracking in the freshly printed 50/50% blend structure, which reveals that degradation and fracture have also started to appear during the printing for these formulations. A lower H_MAHPC content ink containing 25% MAHPC appeared even more amenable to photoprinting, also enabling the production of 150 layered structures and thus centimeter-high parts. The 25/75 percent H_MAHPC/HPC ink clearly outperformed the 50/50 percent formulation in terms of part integrity and part stability while achieving a similar shape fidelity. While the number of layers printed with MAHPC of lower MA content was not specified in prior work [43], rather flat objects were reported, precluding a comparison of shape flexibility in all three dimensions with this work.

Also, we noted that H_MAHPC alone crosslinks into a full 3D network as evidenced by its gel content approaching 100%. This confirms a full cure for neat H_ MAHPC under the printing and UV irradiation conditions used in this study. Additionally, the gel content of samples consisting of 50% HPC and 50% H_MAHPC yielded an insoluble mass of 56.9 ± 3.9%, while the blend of 75% HPC and 25% H_MAHPC showed an insoluble mass of 38.6 ± 5.5%. Thus, the higher the content in photocrosslinkable MAHPC within the blend, the higher the overall gel content of the printed part. For both blends, the gel content deviates positively from the rule of mixture. Namely, with 50% photocrosslinkable MAHPC in the ink, the part exhibits a gel content 7% higher than expected from the rule of mixture. With 25% MAHPC content, deviation from the rule of a mixture of circa 19% is observed. This suggests that during MAHPC crosslinking some HPC is incorporated within the 3D network. This is not so surprising considering HPC’s propensity to photocrosslink in the presence of photocrosslinkers [27]. Expectedly, the more diluted the MAHPC chains are within the HPC neighboring chains (25/75 wt % MAHPC/HPC Ink), the higher the chance to incorporate HPC chains into the crosslinked network and the higher the deviation in gel content from the rule of mixture.

Considering the blending of 25% MAHPC with 75% HPC, which demonstrates excellent curability and stability (Table 1), we propose that a methacrylic group substitution level of 0.5–0.6 could enable successful 3D printing of MAHPC alone under UV light while maintaining good stability.

Finally, thermogravimetric analysis of the printed parts revealed that MA grafting slightly decreases the main degradation temperature of HPC from 388 °C to 378 °C for L_MAHPC and 375 °C for H_MAHPC (Figure S5 and Table S1). Whereas MA grafting has a moderate effect on HPC thermal stability, photocrosslinking has little effect on the main thermal degradation temperatures. Upon crosslinking, however, the onset of degradation for both MA–HPC polymers appears at a much lower temperature, decreasing the T5% by a drastic 100 °C for L-MAHPC and even 180 °C for H-MAHPC (Table S1). The MA grafting reaction and the UV photocrosslinking treatment have, therefore, severely impaired the thermal stability of the MAHPC parts 

## 4. Conclusions

Methacrylated HPC with DS of 1.85 ± 0.4 (L_MAHPC) and of 2.64 ± 0.04 (H-MAHPC), respectively, were evaluated for their suitability in Direct Ink Writing (DIW). MAHPC solutions in ethanol (50% w) exhibited higher zero shear viscosity, stronger shear-thinning and improved shear recovery behaviors than neat HPC solutions, attributes that are all desirable for DIW. These enhancements were ascribed to the formation of a stronger network and fluid structure reversibility thanks to intermolecular interactions of methacryloyl in HPC. Upon UV exposure, the MAHPC inks’ shear storage modulus increased by two orders of magnitude within 30 s, forming a fully networked polymer. For both MAHPCs, the photocrosslinking process was well described by the autocatalytic Sestak–Berggren phenomenological kinetic model. However, neither of the MAHPCs delivered satisfactory 3D parts with DIW, owing to high curing stresses and fracture upon rapid crosslinking. In contrast, blends of HPC with H_MAHPC were successfully processed via DIW, achieving parts with as many as 150 layers and with good shape fidelity and stability. Further work will investigate the potential of MAHPC in lignin/HPC inks as an approach to deliver photocrosslinkable “lignin inks” for fully wood-based 3D parts.

## Figures and Tables

**Table 1:** Characteristics of 3D parts printed from neat HPC, MAHPC inks and their blends.

Photocrosslinkable * Ink Formulations	HPC Ink	25/ 75 wt % MAHPC/HPC Ink	50/50% wt MAHPC /HPC Ink	MAHPC Ink
Upon printing	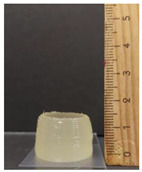	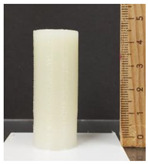	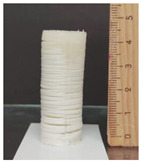	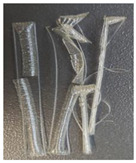
After one week	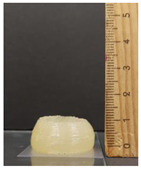	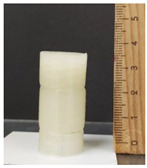	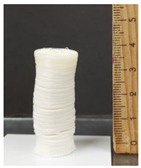	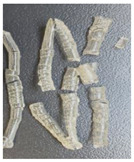
Layers count	50	150	150	1
Shape fidelity	1.2 ± 0.4	0.2 ± 0.02% (z) 0.01 ± 0.01% (both x,y axis)	0.2 ± 0.02% (z) 0.03 ± 0.02% (both x,y axis)	NA
Gel content of printed bars	NA	38.6 ± 5.5%	56.9 ± 3.9%	99.9 ± 0.4%

* All inks include 1% BPO + 0.01% p-Benzoquinone.

## Data Availability

Data are contained within the article or Supplementary Material.

## Supplementary Materials

The following supporting information can be downloaded at: https://www.mdpi.com/article/10.3390/polym17030278/s1, Figure S1: FTIR spectra of acrylic-based chemicals, potential presence in our system, Figure S2: 1H-NMR (a) and 13C-NMR (b) Spectra of neat HPC used for the determinations of hydroxypropyl molar substitution and degree of substitution in HPC, Figure S3: Photograph of the UV-assisted 3D printing setup with Spot P UV-LED light uniform illumination, positioned 30 cm away at approximately 30° angle, Figure S4: Photograph of crack formation in a MAHPC sample during continuous UV light exposure in a rheometer, Figure S5: Unmodified and MAHPC samples pre- and post-photocrosslinking. Table S1: Summary of TGA results [82,83,84].

## Abbreviations

HPC: hydroxypropyl cellulose; MAHPC, methacrylated hydroxypropyl cellulose; H_MAHPC, high degree of substitution methacrylated hydroxypropyl cellulose; L_MAHPC, low degree of substitution methacrylated hydroxypropyl cellulose; MA, methacrylic anhydride; MAA, methacrylic acid; DMAP, 4-Dimethylaminopyridine; SpeedCure BPO or BPO, phenyl bis(2,4,6-trimethylbenzoyl)-phosphine oxide; Chromium(III) acetylacetonate, Cr(acac)3; DIW, direct ink writing; DS, degree of substitution; MS, molar substitution degree; LVE, linear viscoelastic range.
