# Supplementary material for: Direct Ink Writing and Photocrosslinking of Hydroxypropyl Cellulose into Stable 3D Parts Using Methacrylation and Blending
Source: Polymers (Basel). 2025 Jan 22;17(3):278. doi: 10.3390/polym17030278 (PMC11821224; doi:10.3390/polym17030278)
Supplement: Supplementary file 1 [file polymers-17-00278-s001.zip › polymers-3385670-supplementary.pdf]

SUPPLEMENTARY INFORMATION

Enabling Photoprinting of Hydroxypropyl Cellulose by Direct Ink Writing Through Methacrylation and Blending

Mehmet-Talha Yapa [1,2], Gopakumar Sivasankarapillai [1,2], Jacques Lalevée [3,4], Marie-Pierre Laborie [1,2,5] *

1. Chair of Forest Biomaterials, Institute of Earth and Environmental Sciences, Faculty of Environment and Natural Resources, University of Freiburg, Werthmanstr. 6, 79085 Freiburg im Breisgau, Germany
2. Freiburg Materials Research Center, Stefan-Meier-Straße 21, 79104 Freiburg im Breisgau, Germany
3. Université de Haute Alsace, CNRS, IS2M UMR 7361, Mulhouse 68100, France
4. Strasbourg University, 4 Rue Blaise Pascal, 67000 Strasbourg, France
5. Strasbourg University, CNRS, Institut Charles Sadron UPR22, 23 rue du Loess, 67000 Strasbourg, France

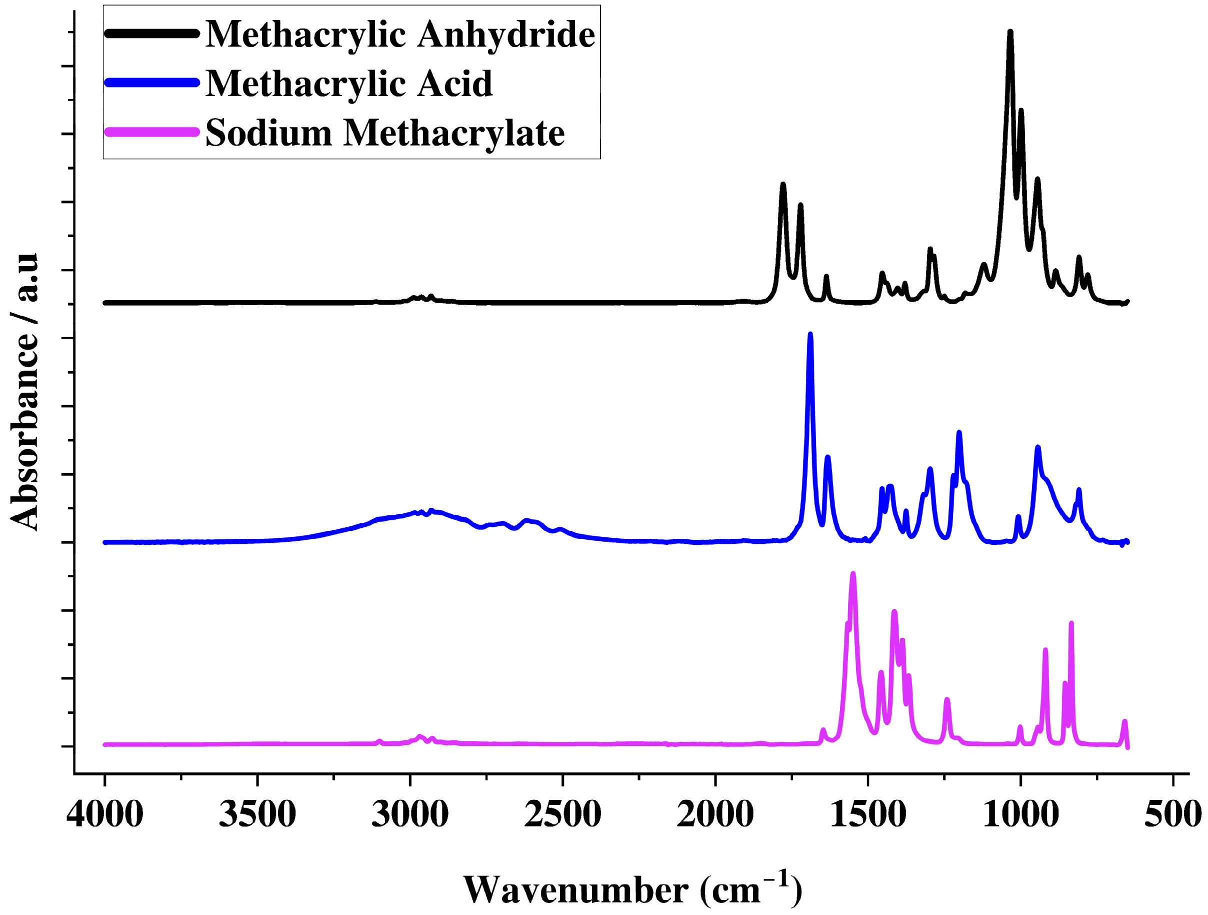

Figure S1. FTIR spectra of acrylic-based chemicals, potential presence in our system

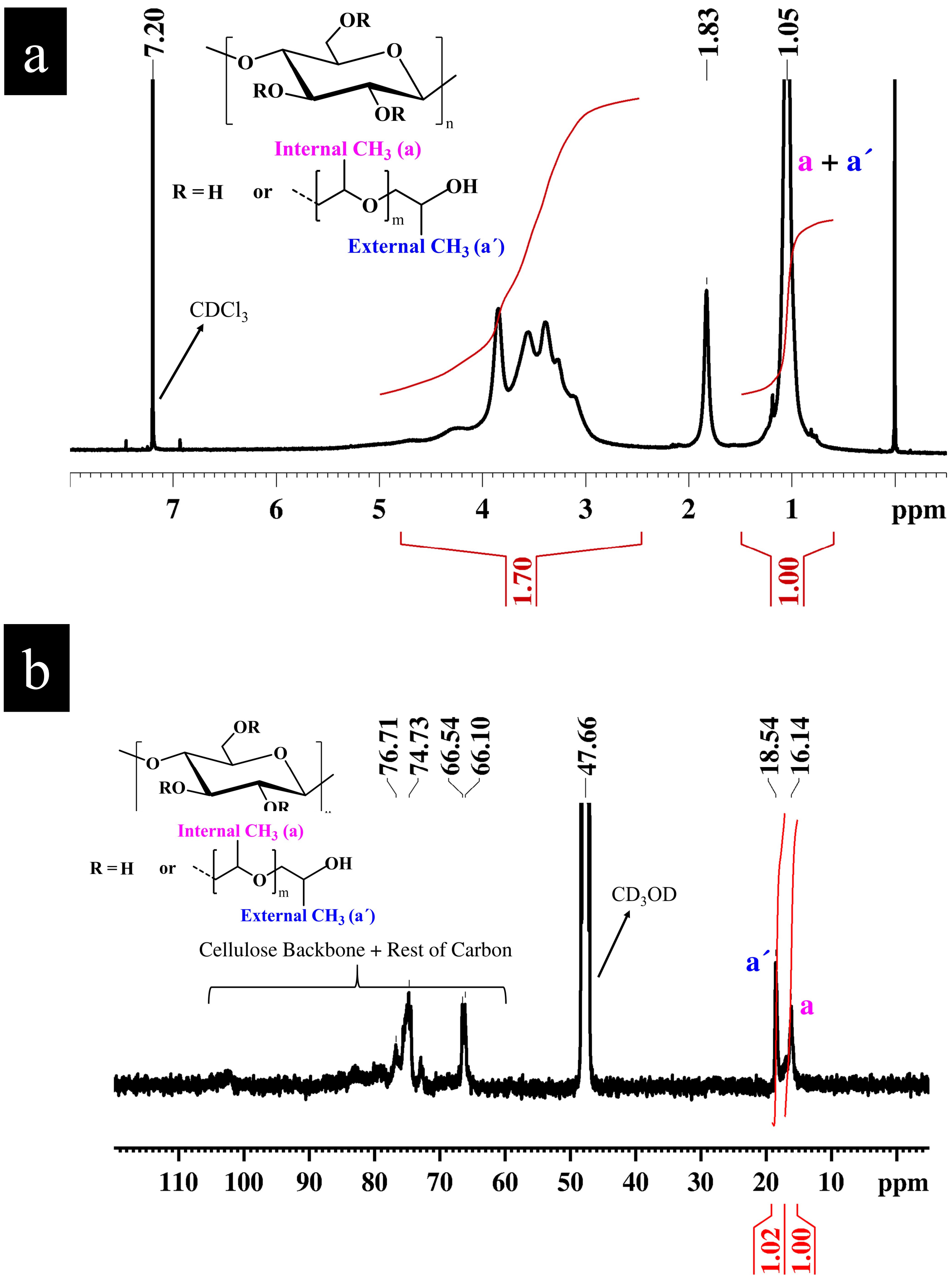

Figure S2. ^{1}H-NMR (a) and ^{13}C-NMR (b) Spectra of neat HPC used for the determinations of hydroxypropyl molar substitution and degree of substitution in HPC

The hydroxypropyl molar substitution degree (MS) was computed according to established protocols [61, 62] and yielded a value of 4.76.

$$\mathrm{MS} = \frac{10}{3 \times \left[\frac{\int(2.5 - 4.85\, ppm)}{\int(0.7 - 1.5\, ppm)} - 1\right]}$$

The hydroxypropyl degree of substitution degree (DS) was then computed according to established protocols [82] relying on additional signal intensities from ^{13}C NMR spectra as: For calculating DS, the following referenced formula is applied:

$$\mathrm{DS} = \frac{\mathrm{MS\ from\ H-NMR}}{\left(1 + \frac{\int \mathrm{Internal{-}CH_3\ signal\ (a)\ of\ HPC\ from\ C-NMR\ (15.2 - 17.2\ ppm)}}{\int \mathrm{External{-}CH_3\ signal\ (a')\ of\ HPC\ from\ C-NMR\ (17.2 - 19.2\ ppm)}}\right)}$$

A DS of 2.4 was obtained in alignment with previous reports for the same HPC (from a different supplier) of a DS of 2.5 and a MS of 4 [53]. Validation of this DS was obtained from the ^{1}H NMR of highly methacrylated HPC [39, 62, 63, 83] since distinct resonances for the internal and external CH3 of the hydroxypropyl independently enabled quantification of the internal /external CH_3 ratio at 1/103.

Determination of the degree of methacrylate grafting of MAHPC from ^{1}H NMR Spectroscopy

The degree of substitution for methacrylic groups is obtained from ^{1}H NMR as:

$$\mathrm{DS} = \frac{(\int \mathrm{CH2} = \mathrm{CH}- \text{ signal of MAHPC } (\mathrm{c} + \mathrm{c}') \text{ between } 5.3 - 6.1 \text{ ppm from H} - \mathrm{NMR}}{\text{Number of AGUs } \times 2}$$

The number of AGUs is computed from the ratio of methyl signal and the MS of HPC [64, 84] as:

$$\text{Number of AGUs} = \frac{\int -\mathrm{CH_3} \text{ signal of HPC } (\mathrm{a} + \mathrm{a}') \text{ between } 0.7 - 1.5 \text{ ppm from H} - \mathrm{NMR}}{\text{MS of HPC } \times 3}$$

The DS values for methacrylic groups were determined to be 1.85 ± 0.04 for L_MAHPC and 2.64 ± 0.04 for H_MAHPC, respectively.

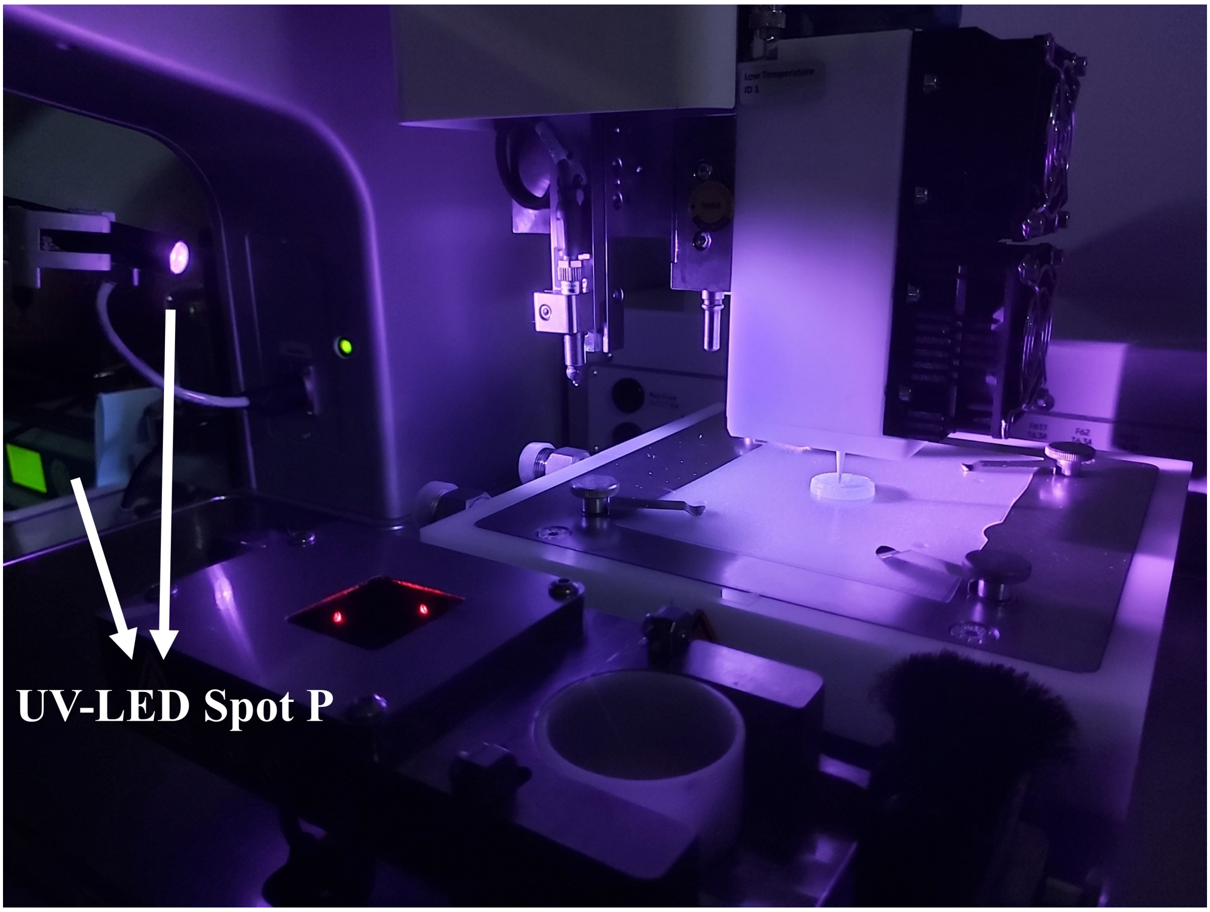

Figure S3. Photograph of the UV-assisted 3D printing setup with Spot P UV-LED light uniform illumination, positioned 30 cm away at approximately 30° angle

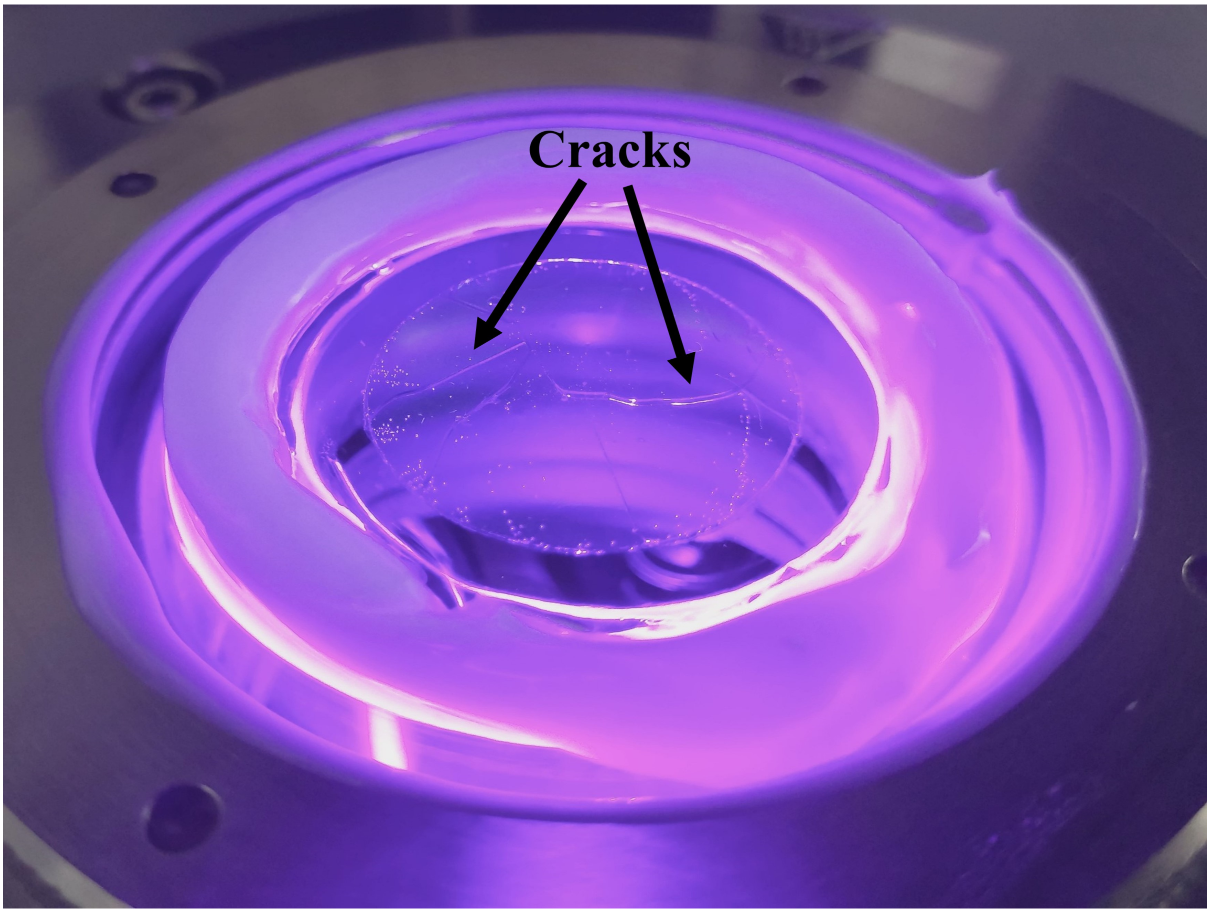

Figure S4. Photograph of crack formation in a MAHPC sample during continuous UV light exposure in a rheometer

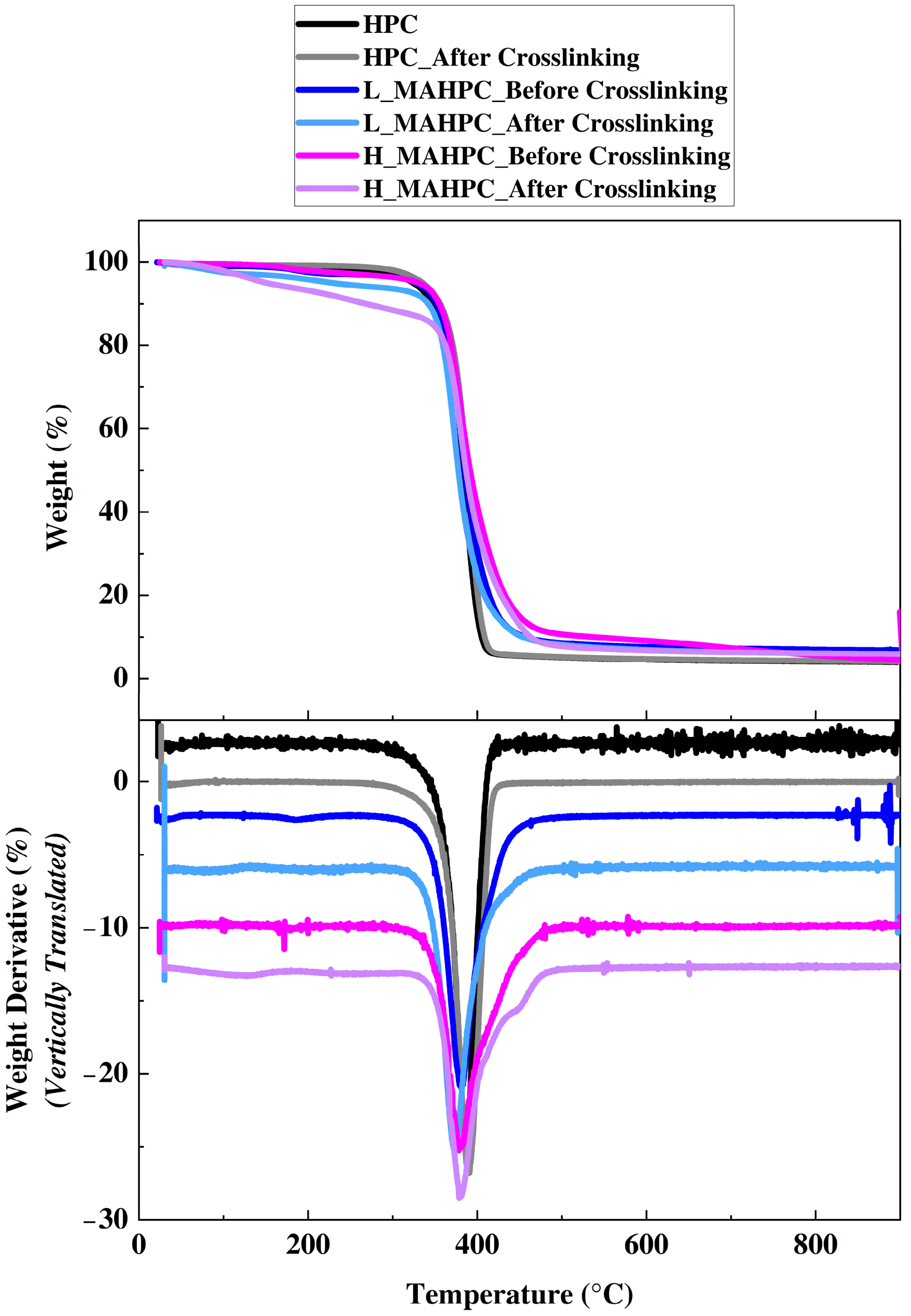

Figure S5. Unmodified and MAHPC samples pre- and post-photocrosslinking

Table S1. Summary of TGA results

	HPC	HPC photocrosslinked *	L_MAHPC	L_MAHPC photocrosslinked *	H_MAHPC	H_MAHPC photocrosslinked *
Water Content (%)	1.6 ± 0.1	1.3 ± 0.3	1 ± 0.1	0.29 ± 0.2	0.6 ± 0.1	2.3 ± 1.9
T5% (°C)	323.3 ± 19.4	334.1 ± 33.7	327.5 ± 22.3	226 ± 23.5	329.7 ± 6.9	150.3 ± 8.8
Tdegradation (°C)	388 ± 2	390 ± 4.9	377.8 ± 5.2	373.5 ± 12.7	375.1 ± 4.2	378.9 ± 7.7
Residual Mass (%)	4 ± 0.1	5 ± 2.1	6.3 ± 0.7	6.1 ± 4.5	4.2 ± 0.2	9.2 ± 2.4

* photocrosslinking activated with1% BPO + 0.01% p-benzoquinone
